# Schottky barrier formation and band bending revealed by first- principles calculations

**DOI:** 10.1038/srep11374

**Published:** 2015-06-12

**Authors:** Yang Jiao, Anders Hellman, Yurui Fang, Shiwu Gao, Mikael Käll

**Affiliations:** 1 Department of Applied Physics, Chalmers University of Technology, Göteborg, SE-412 96, Sweden; 2 Beijing Computational Science Research Center, Beijing, 100094, China

## Abstract

The formation of a Schottky barrier at the metal-semiconductor interface is widely utilised in semiconductor devices. With the emerging of novel Schottky barrier based nanoelectronics, a further microscopic understanding of this interface is in high demand. Here we provide an atomistic insight into potential barrier formation and band bending by *ab initio* simulations and model analysis of a prototype Schottky diode, *i.e.*, niobium doped rutile titania in contact with gold (Au/Nb:TiO_2_). The local Schottky barrier height is found to vary between 0 and 1.26 eV depending on the position of the dopant. The band bending is caused by a dopant induced dipole field between the interface and the dopant site, whereas the pristine Au/TiO_2_ interface does not show any band bending. These findings open the possibility for atomic scale optimisation of the Schottky barrier and light harvesting in metal-semiconductor nanostructures.

The presence of a Schottky barrier (SB) between a semiconductor and a metal is of paramount importance to numerous application fields, including electronics^1^, photovoltaics^2^,^3^ photocatalysis^4^,^5^,^6^ and gas sensors^7^,^8^,^9^ Schottky barrier physics has been a subject of intense investigation for several decades, but has recently received renewed substantial attention in two areas: i) the emergence of novel Schottky devices in plasmonics for photocurrent generation, photo detection and solar light harvesting^5^,^10^,^11^ and ii) the development of quantum-scale metal-semiconductor structures, pushed by the ever present need to further minimise and optimise electronic devices^12^,^13^,^14^,^15^,^16^,^17^ Continued development of these areas could be greatly facilitated by an atomistic understanding of SB-based processes.

The quantum transmission of electrons or holes across the SB is determined by two quantities: the barrier height and, more importantly, the decay length of the band bending. Together, these quantities determine the probability of transmission and the energy distribution of hot carriers across the metal-semiconductor interface. The conventional SB model assumes a uniform charge depletion region on the semiconductor side and a charge accumulation layer localised at the interface^18^,^19^ resulting in a parabolic bending of the semiconductor bands. The decay length of the band bending has been believed to be on the order of 10 nanometers for typical dopant concentration (10^19^ cm^−3^). However, recent advances in nanotechnology^12^,^20^,^21^ which has made it possible to control and characterise the SB at the nanometer scale, have revealed important deviations from predictions made from the homogeneous Schottky barrier height (SBH) model^14^. Instead, the results, which depend on materials properties, dopant compositions and concentrations, have been qualitatively interpreted in the inhomogeneous SBH model^22^,^23^ although the current lack of a complete atomistic picture severely limits the possibility to achieve a quantitative understanding of SB formation. In particular, conventional models all assume a uniform dopant distribution, and it remains unclear how the composition and atomic structure of the semiconductor affect the electronic structure, i.e., barrier height and band bending, on the atomic level.

Here, we report first-principles calculations of a prototype Schottky diode, *i.e.*, the Au/TiO_2_ interface. A substitutional niobium dopant (*Nb*_*Ti*_) was introduced to model discrete defects in the n-type semiconductor. The concentration and spatial distribution of the *Nb*_*Ti*_ can be controlled experimentally^24^,^25^,^26^,^27^ and Nb dopants are known to induce small lattice relaxations^28^ which makes this particular system suitable for atomistic simulations. We found that the pristine Au/TiO_2_ interface has a relatively large barrier height, but shows no band bending. The band bending and decay length is instead determined by the precise locations of the dopant. Our results also show that the band bending is inhomogeneous and highly localised to the defect region. We calculated the dopant position dependent barrier height and show that it can be qualitatively understood by the deep level (DL) barrier model^29^. Our results reveal the origin and nature of inhomogeneity of the SBH and shed light on the mechanisms of electron transmission across the metal-semiconductor interfaces.

## Results

**Figure 1:**
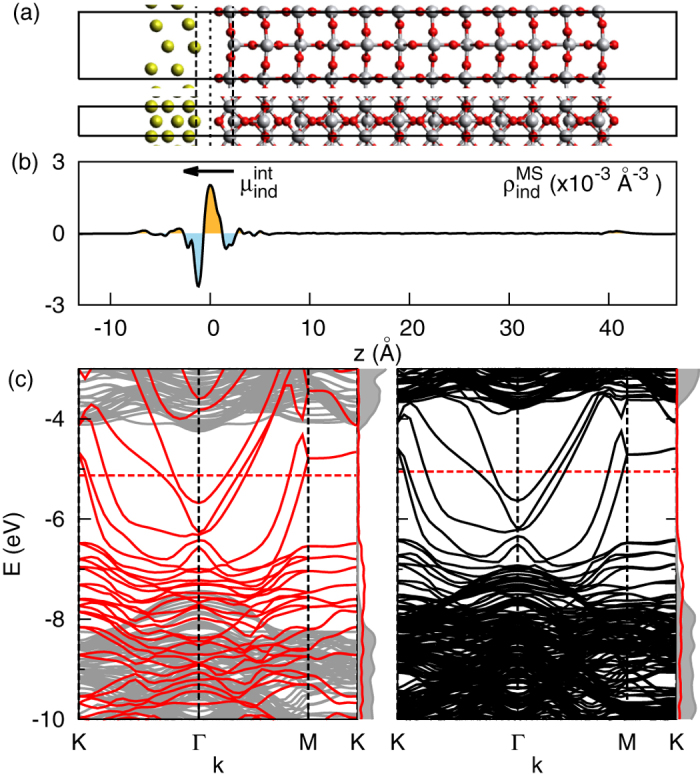
The geometry and electronic structure of the pristine interface show that the TiO_2_ band shifts up owing to the influence of the induced interface dipole. (**a**) Schematic of the Au (112) and TiO_2_ rutile (110) interface used in the calculations. The boxes show the cell employed in the periodic boundary calculations. (**b**) The plane (parallel to the slab) averaged induced charge density. Red and blue indicate charge accumulation and depletion. The induced charge density gives rise to a dipole at the interface. (**c**) The band structure of isolated (left) and combined (right) Au and rutile TiO_2_ slab. The red dashed line is the Fermi level. The side columns show the density of states (DOS) of the Au (red) and TiO_2_ (gray) components. The TiO_2_ bands shift up by 0.4 eV owing to the contact with Au.

In order to explore the effect of chemical composition on the SB, a large number of different atomic structures were calculated based on the lattice alignment and orientation of Au nanoparticles on thin film rutile (110) TiO_2_^30^. In epitaxial growth, the Au[110] close-packed direction is always found to be parallel to the TiO_2_[001] direction with a lattice mismatch as small as 0.4%. Different epitaxial layers are obtained by rotating around the [110] axis. In the following, we focus on the Au(112)/TiO_2_(110) interface, which has been observed after high temperature deposition^31^. In this case, one Au atom is located on top of Ti_5*c*_ in the rutile TiO_2_(110) surface (Fig. 1a)^31^. We modelled the interface in a slab geometry using a 12 O-Ti-O trilayers 40 Å thick) in contact with 3 layers (5 Å thick) of Au(112). The periodic slabs were separated by a 15 Å thick vacuum region (Fig. 1a), and dipole correction was used to alleviate the image interactions. The workfunction of the Au slab (*ϕ*_*m*_) and the electron affinity of the TiO_2_ slab (*χ*_*s*_) were calculated to be 5.13 eV and 4.27 eV, which is in good agreement with experimental values (5.2 eV^32^ and 4.3 eV^33^ respectively). The Nb-dopant was introduced by replacing one of the Ti atoms in the lattice (*Nb*_*Ti*_). In addition, the effect of changing the concentration of the Nb-dopants was checked by increasing the surface unitcell to (1×2) and (2×3) using a 5-layer TiO_2_ slab.

### Step like barrier at pristine interface

The electronic structure of the pristine interface is shown in Fig. 1. The lower panels show the band structures of the isolated Au and TiO_2_ slabs (left) and the interface formed upon contact (right). The interface band structure reveals a rigid upward shift of about 0.5 eV for both the valence and conduction bands of TiO_2_, while the Fermi level and Au bands change by less than 0.1 eV. This difference is caused by charge polarisation, as shown in panel (b). We analysed this effect using the contact induced charge density 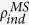 and doping induced charge density 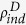 defined as:


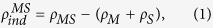



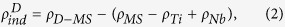



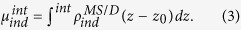


Here, *ρ*_*MS*_ and *ρ*_*D*_*-*_*MS*_ are the charge densities of the undoped and doped Au/TiO_2_ interfaces, respectively, *ρ*_*M*_ and *ρ*_*S*_ are those of the isolated metal and semiconductor slabs, while *ρ*_*Ti*_ and *ρ*_*Nb*_ are the charge densities of the free atoms. The areal density of the interface induced dipole 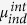 was calculated by integration over an interface range of 5 Å and found to be 
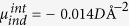
. The negative sign means that the dipole points from TiO_2_ to Au (Fig. 1b). The charge transfer between Au and undoped TiO_2_ is found to be less than 0.05 e/cell, as shown by a Bader analysis. The induced dipole results in a SBH of 1.26 eV, which is 0.4 eV larger than the difference between the work function and the electron affinity of the isolated Au and TiO_2_ slabs. The band gap of TiO_2_ is 3.3 eV, that is the same as in the bulk calculation. The interface position (*z* = 0) was chosen at the electronic potential maximum between the TiO_2_ and Au layers. Importantly, the conduction band edge is found to be flat throughout the semiconductor slab (see Supplementary Fig. S1 online).

### Dopant induced band bending

**Figure 2:**
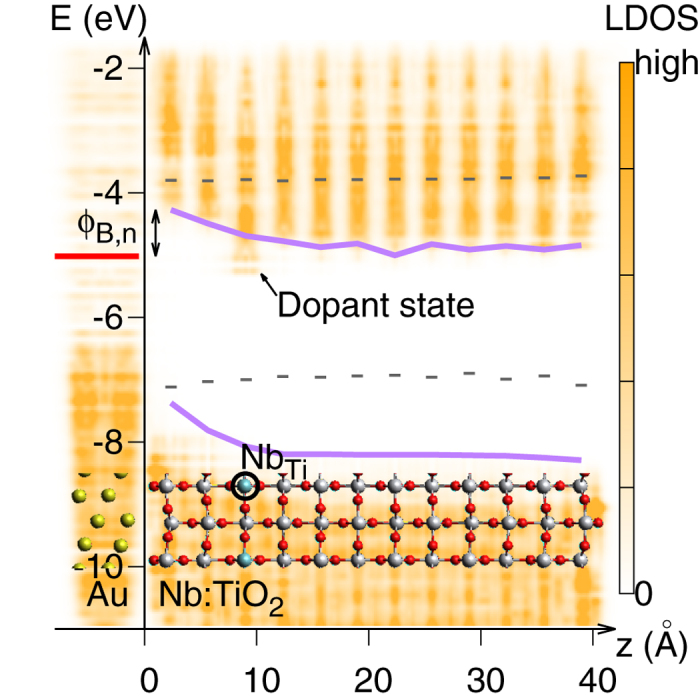
Illustration of Schottky barrier formed at a Au/Nb:TiO_2_ interface. The color map shows the local density of states (LDOS), as obtained from the DFT+U calculations, along the direction normal to the interface. The Schottky barrier height for n-type doping (Ф_*B,n*_) is defined as the energy difference between the Fermi energy (red line) and the conduction band minimum (upper purple line) at the interface. The Nb-dopant is marked as *Nb*_*Ti*_ in the inserted structure plot. The dopant state below the bottom of the conduction band traps most of the excess electron donated by *Nb*_*Ti*_. The band edges of the pristine TiO_2_ are also shown (gray dashed lines). The band bending is caused by the dopant induced charge polarisation.

The results above show that the pristine TiO_2_/Au interface does not exhibit any band bending in the semiconductor region, and we therefore hypothesised that the atomic scale SBH inhomogeneity and band bending instead are caused by the dopant. Figure 2 shows a typical case with a Nb-dopant placed two layers beneath the interface. Here the SBH (Ф_*B,n*_) is defined as the energy difference between the Fermi level (*E*_*F*_) and the conduction band minimum (CBM) of the TiO_2_ layer at the interface. We found that both the potential profile and the SBH critically depend on the position of the dopant. When the *Nb*_*Ti*_ is located at the contacting layer, the defect state density is high enough to pin the Fermi level, which is very close to the bottom of the conduction band, as found in previous studies^34^. In this case, the SBH is significantly reduced, and the band bending is the same as for the pristine case, i.e. it is essentially flat. However, as the dopant is moved away from the interface, the dopant induced charge is distributed nonuniformly between the interface and the dopant, leading to a lowering of the potential towards the dopant, i.e., a clear band bending. This trend starts from the second layer (dopant-interface distance *d*_*D*_ = 5.9 Å) and becomes more prominent as the dopant moves to the other end of the slab at 12*th* layer (*d*_*D*_ = 39.2 Å). Thus, our results clearly show that the local dopant confines the SBH and dictates the band bending.

**Figure 3:**
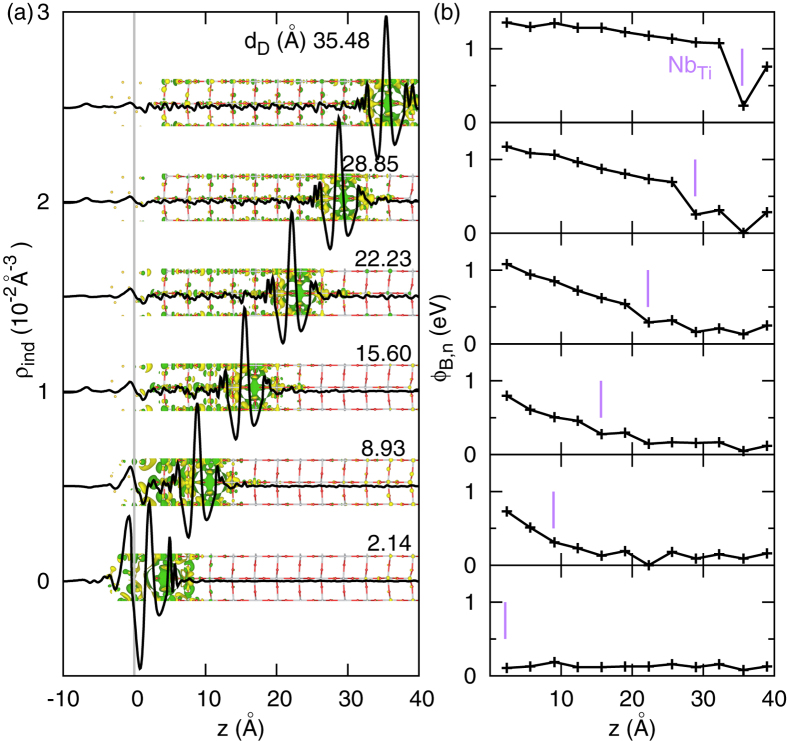
The dopant-interface distance (*d*_*D*_) dependence of the induced charge and band bending. (**a**) *Nb*_*Ti*_ induced charge density. The curves are the plane averaged value and the colour inserts are the isosurface with isovalue = 10^−3^Å^−3^. Yellow (+), Green (–). The induced interface dipole is inversely proportional to *d*_*D*_. (**b**) Band bending in TiO_2_. The points are the local conduction band minimum (CBM) in each layer in relation to the Fermi energy. The vertical bar indicates the position of Nb-dopant in each calculation. The TiO_2_ band shifts up while approaching the interface.

The variation in charge and potential with dopant-interface distance are analysed in Fig. 3. With the Nb-dopant positioned between the second layer and the fifth layer, the band bending is nearly parabolic. This is consistent with the uniform dopant SB model. However, it becomes essentially linear if the Nb-dopant is located beyond the fifth layer, i.e. *d*_*D*_ > 16 Å. The magnitude of the induced interface dipole is found to be inversely proportional to the dopant-interface distance, 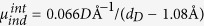 (see Supplementary Fig. S2 online), and the dipole points from Au to Nb:TiO_2_, while the induced dipole around the dopant points in the opposite direction. The charge transfer between the metal and the semiconductor slabs was found to be negligible for the pristine interface as well as for the doped cases (0.07 e/cell in case of interfacial *Nb*_*Ti*_ , and less than 0.02 e/cell at other doping positions). The excess electron contributed by the Nb-dopant is instead mainly distributed on the Nb atom and the neighbouring Ti atoms along the [001] direction. This localized charge distribution is consistent with previous DFT+U calculations^28^ and STM experiments^35^. Further Fig. 3(b) shows that the CBM of the layer containing the dopant was pinned to the energy *E*_*F*_ + ζ eV, where ζ is in the range of 0.2 ∼ 0.5 eV and varies with *d*_*D*_. The same shift was observed for the Ti 4 s semi-core states (see Supplementary Fig. S3 online). Thus, we can conclude that dopant induced charge polarization gives rise to a dipole field that is mainly responsible for the band bending. We can further conclude that the SBH and band bending are highly inhomogeneous (locally determined) and strongly dependent on the dopant-interface distance within a range of a few nanometers.

### Schottky barrier height reduction by dopant

**Figure 4:**
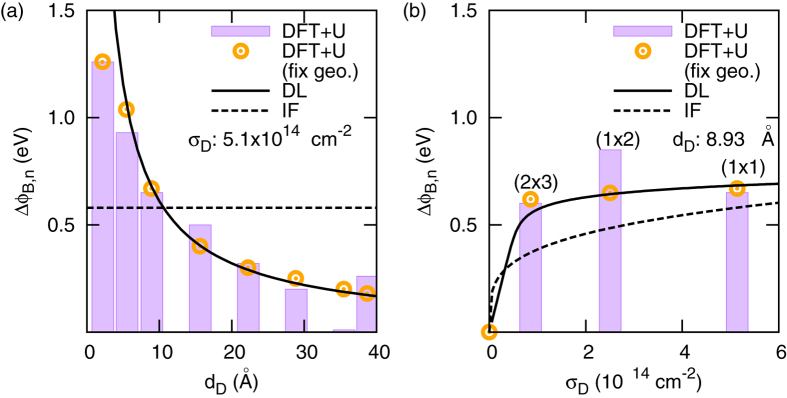
Schottky barrier height reduction (ΔФ_*B,n*_) as a function of (**a**) dopant-interface distance (*d*_*D*_) and (**b**) dopant areal concentration (*σ*_*D*_). The histogram show the DFT+U results obtained with full geometry optimisation. The circles are calculations with atomic coordinates fixed at the pristine interface geometry. The solid lines are calculated using the DL model (Eq. 5) while the dashed lines are obtained from the uniform dopant Schottky model including an image force (IF) correction (Eq. 4).

The atomic picture described above is in sharp contrast to the conventional SB picture, which does not include any dopant position dependence as it assumes that the band bending is caused by the electrostatic potential generated by uniform ionised defects in the depletion layer^18^,^19^. The conventional potential profile consists of a parabolic term plus an image charge correction term^18^,^36^. The image force (IF) decreases the SBH by^19^:


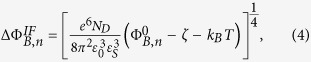


where *e* is the charge of the electron, *N*_*D*_ is the dopant concentration, *ε*_0_ and *ε*_*S*_ are the vacuum permittivity and relative dielectric constant of semiconductor, respectively, 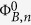 is the SBH in the absence of image charge correction, *ζ* is the energy difference between the CBM and the Fermi level, *k*_*B*_ is Boltzmann’s constant and *T* is the temperature. Using the geometry from our first-principles calculations, i.e. one *Nb*_*Ti*_ in the long (1×1×12) cell, we have *N*_*D*_ = 1.3×10^21^ cm^−3^. The depletion layer width is found to be 10 Å (25 Å) with *ε*_*S*_ = 10 (*ε*_*S*_ = 60). The SBH reduction is then 0.58 eV for *ε*_*S*_ = 10 (0.15 eV for *ε*_*S*_ = 60) (see Supplementary Fig. S4 online), which deviates from the calculated SBH reduction (Fig. 4), and does not explain the dependence on the dopant position (Fig. 3). This qualitative and quantitative discrepancy between the SBH prediction obtained from the uniform dopant Schottky model and our results clearly indicate the importance of an atomistic description of the interface.

We now compare the DFT+U results with the alternative deep level (DL) model^29^ which was developed to specifically incorporate the local barrier profile near the interface. The DL model assumes point charge donors with energy (*E*_*DL*_) below the CBM. The SBH reduction depends on the charge donor-interface distance (*d*_*DL*_) and the areal dopant density (*σ*_*DL*_) according to^29^


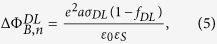


where 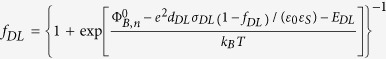 is the Fermi-Dirac distribution function of the DL states. We extracted the relevant parameters from the DFT+U calculations (Fig. 3): 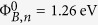 is the SBH without DL states, *a* *=* *5* Å is the characteristic width of the interface, *E*_*DL*_ = 0.2 ev is the DL energy below the CBM, and we set *ε*_*S*_ = 10 and *T* = 1000 *K*. The resulting SBH obtained from the DL model is plotted in Fig. 4 and is found to be in good agreement with our first-principles calculations, except for the regions closest to the boundaries. To pin down the origin of this discrepancy, we also performed calculations for a case where the atomic structure of the Nb-doped slab were fixed at the pristine interface structure position (DFT+U (fix geo.) in Fig. 4). The DL model then captures the main dopant dependent SBH feature extremely well, the only exception is the case when the dopant is located at the interfacial layer where the DL model is not applicable.

## Discussion

In conclusion, first-principles calculations of Au/TiO_2_ interfaces show that the SBH is tuned by interface dipoles induced by contact and dopant. The local barrier profile, i.e. the band bending, shift almost linearly between the interface and the dopant layer. The barrier width is a-few-layer thick and depends on the dopant position. The reported experimental SBH for Au/TiO_2_ interfaces lies in the range 0.9 ~1.2 eV^8^,^37^,^38^. Given that these measurements are macroscopically averaged, our calculations are in very good agreement with experiment. In contrast to the uniform dopant Schottky model, the DL model is able to account for the SBH reduction variation with dopant position. The conclusion and overall picture emerging from the present study should be generally applicable and highly relevant also to other metal-semiconductor systems. As such, they can serve as a basis and reference to further studies of internal electron emission and hot-carrier transport across metal semiconductor interfaces. In light of the rapid development of layer-controlled molecular beam epitaxy^39^ the results open up the possibility for atomic scale engineering and optimisation of novel SB-based devices.

## Methods

All the calculation in this work was done using the Vienna Ab initio Simulation Packages (VASP)^40^,^41^,^42^,^43^ with the projector augmented wave (PAW) method^44^,^45^ and the PBE^46^,^47^ exchange-correlation functional in the generalised gradient approximation (GGA). The Coulomb correlation of the Ti 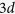 orbitals were treated in the GGA+U scheme^48^ with an effective on-site Coulomb repulsion U = 10 eV. The plane wave cut-off energy was set to 400 eV. A Monkhorst-Pack k-point mesh of 5×11×1 centred at the Γ point was used for the Au/TiO_2_ rutile (110) interface supercell. A more condensed k-sampling (9×21×1) was adopted when extracting the density of states (DOS). The geometry optimisations were done on the GGA level. All of the atoms were relaxed in the geometry optimisation processes, and were considered to be relaxed when the maximum forces on each atom was smaller than 0.01 eV/Å. The calculated lattice constants of TiO_2_ (rutile) and Au (fcc) are *a* = 4.64 Å, *c* = 2.96 Å, *u* = 0.305 and *a* = 4.17 Å, respectively, in good agreement with experimental values^30^.

In the DFT+U calculations, the on-site Coulomb repulsion U parameter for Ti 3d orbital was carefully calibrated by calculating the bandgap of pristine rutile TiO_2_ and comparing with results from both G_0_W_0_ calculations and experimental measurements. The calculations were performed using a cell with 6 atoms and a 9×9×11 Monkhorst-Pack k-point sampling. The parameter was determined to be 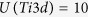 eV (see Supplementary Table S1 online). Furthermore, with this value the electron affinity of 12-layer TiO_2_ rutile slab was found to be 4.27 eV, which also agrees well with the experimental value of 4.33 eV^33^.

The *U*(*Nb*4*d*) was then determined by keeping the *U*(*Ti*3*d*) fixed at 10 eV and calculating the dopant state with *U*(*Nb*4*d*) varied from 0 eV to 5 eV. A supercell of 13.13×8.88×6.57 Å^3^ along [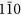], [001] and [110] directions was used. The Nb dopant occupies one of the host Ti sites within the supercell^24^, thus forms Ti_1–*x*_Nb_*x*_O_2_ with *x* = 0.042, which is equivalent to our 12-layer TiO_2_ calculations (a dopant concentration of 1.3×10^21^ cm^−3^ and 4.7 wt%). Higher concentration of Nb-dopant was also calculated using the same supercell but with more Ti atoms replaced by Nb. While *U*(*Nb*4*d*)≤3, the extra charge carrier distribution (see Supplementary Table S2 online) and states blow the CBM (see Supplementary Fig. S5 online) were not sensitive to the varied value of *U*(*Nb*4*d*). A dopant state locates at 1.3 ∼ 1.5 eV below the CBM and about 0.5 eV below the Fermi level, and the extra charge was trapped on neighbour Ti atoms along [001] direction. The calculated DOS below the CBM was found compatible with x-ray photoemission spectra (XPS)^24^ (see Supplementary Fig. S6), and the charge transfer to the neighbouring Ti atoms along [001] direction created a polaron in agreement with previous studies^28^. For U(Nb4d) ≥ 4 eV, the dopant state upshifts to combine with the conduction band edge, which results in markable changes in the charge distribution (see Supplementary Table S2 online). Based on the consideration of the defect state energy distribution and charge carrier localisation, *U*(*Nb*4*d*) = 0 is sufficient in this work.

## Additional Information

**How to cite this article**: Jiao, Y. *et al.* Schottky barrier formation and band bending revealed by first-principles calculations. *Sci. Rep.* **5**, 11374; doi: 10.1038/srep11374 (2015).

## Supplementary Material

Supplementary Information
