# Supplementary material for: Schottky barrier formation and band bending revealed by first- principles calculations
Source: Sci Rep. 2015 Jun 12;5:11374. doi: 10.1038/srep11374 (PMC4464327; doi:10.1038/srep11374)
Supplement: Supplementary Information [file srep11374-s1.pdf]

Supplementary information: Schottky barrier formation and band bending revealed by first-principles calculations

Yang Jiao[1], Anders Hellman[1,*], Yurui Fang[1], Shiwu Gao[2,*], and Mikael Käll[1]

[1]Department of Applied Physics, Chalmers University of Technology, Göteborg, SE-412 96, Sweden
[2]Beijing Computational Science Research Center, Beijing, 100094, China
[*]anders.hellman@chalmers.se
[*]swgao@csrc.ac.cn

Supplementary Figures

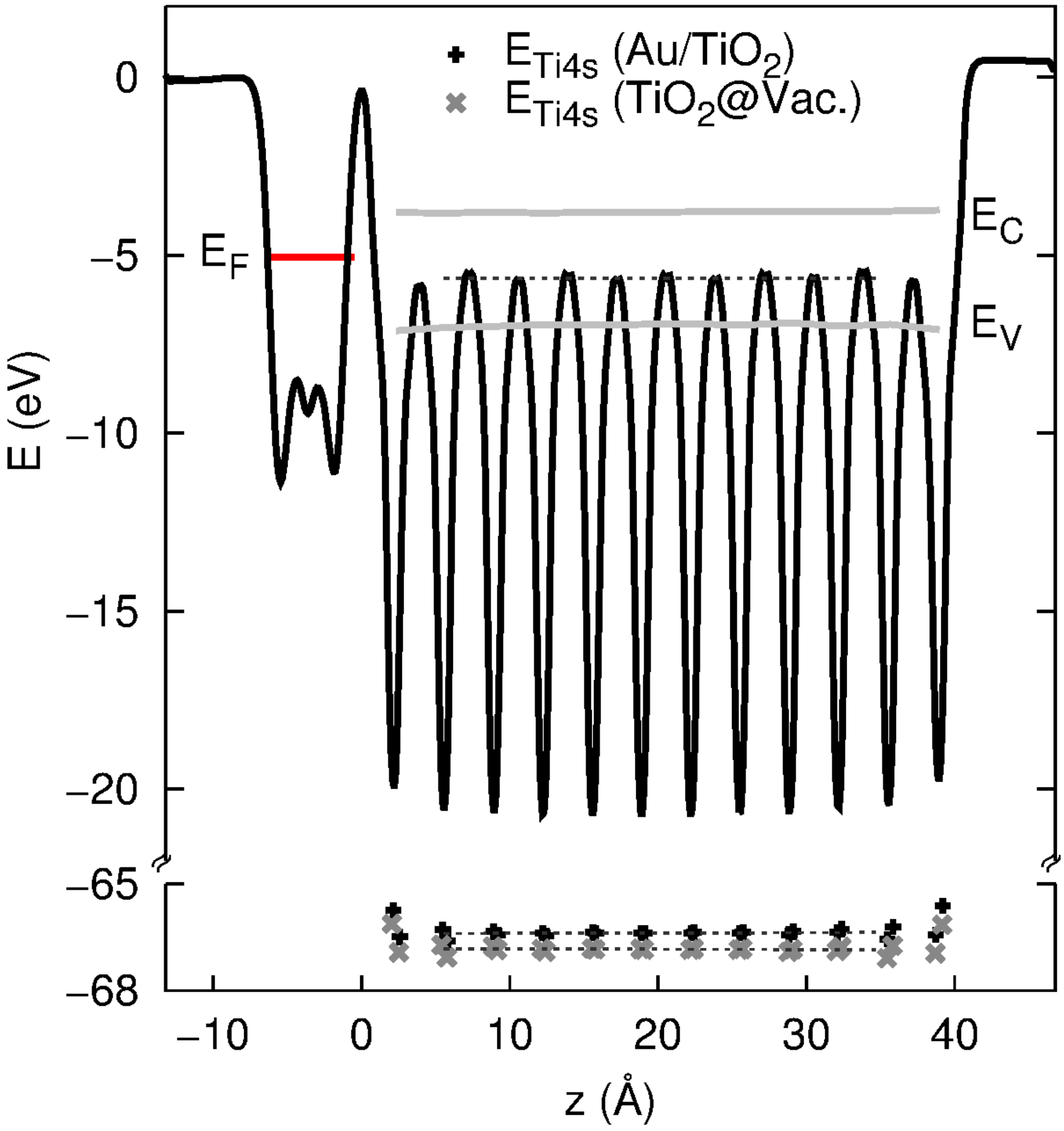

Supplementary Figure S1. The step-like barrier at the pristine Au/TiO_2 interface. The solid curve shows the plane averaged electrostatic potential of the Au/TiO_2 slab. The grey lines are the connection of the band edge of each TiO_2 layer. The dots are the energy level of Ti 4s electrons in free standing and Au attached TiO_2 slabs. The interface to the Au slab upshifts the energy level of TiO_2 slab by 0.4 eV as a result of the interface electron polarisation. The dotted lines are guide lines to show the potential flatness in the interior of TiO_2 slab.

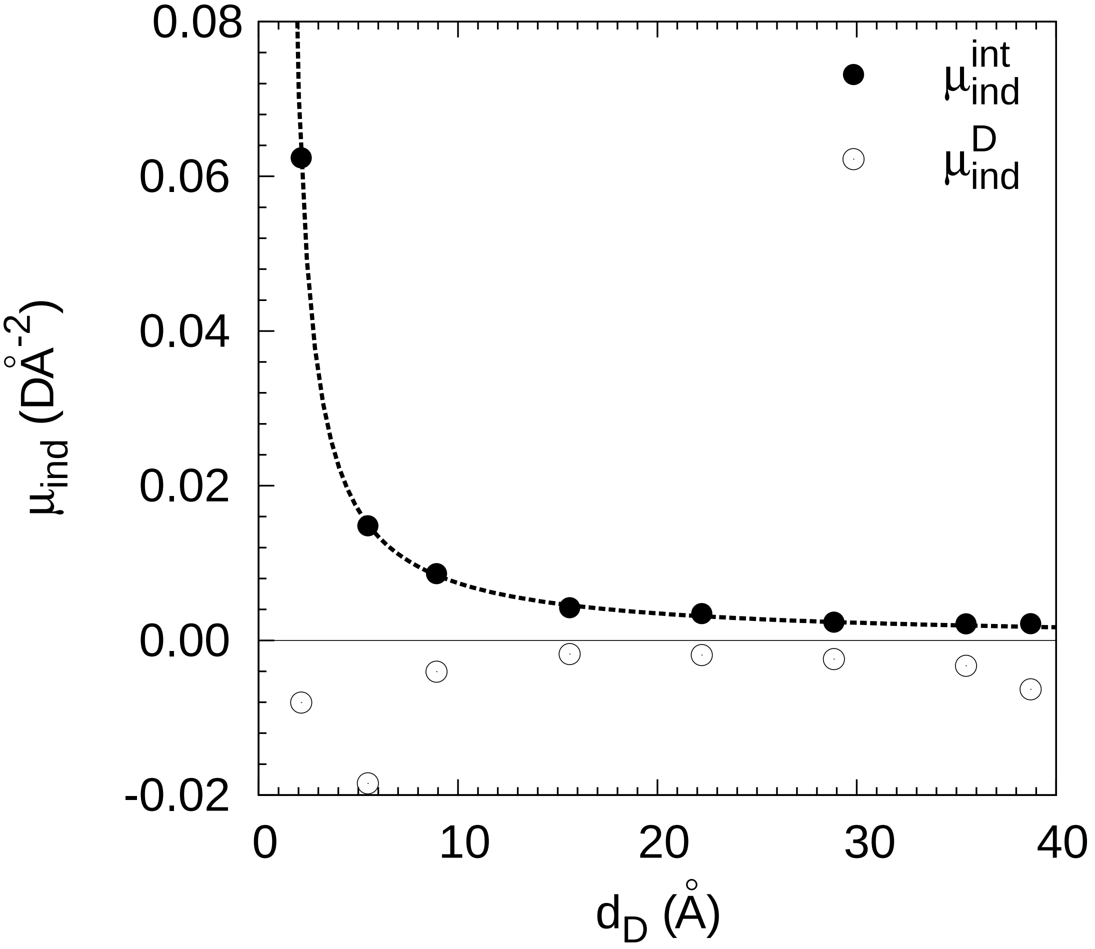

Supplementary Figure S2. The induced dipole at the interface (μ_{ind}^{int}) and around the Nb dopant (μ_{ind}^{D}) as a function of dopant interface distance (d_D). The magnitude was calculated by integrating of Eq. 3 over the interface range and dopant layer. The induced dipole at the interface (μ_{ind}^{int}) was found to be in reverse proportional to the dopant interface distance (d_D). The dotted curve is fitted by $\mu_{ind}^{int} = 0.066\ \mathrm{D\AA}^{-1}/(d_D - 1.08\ \mathrm{\AA})$.

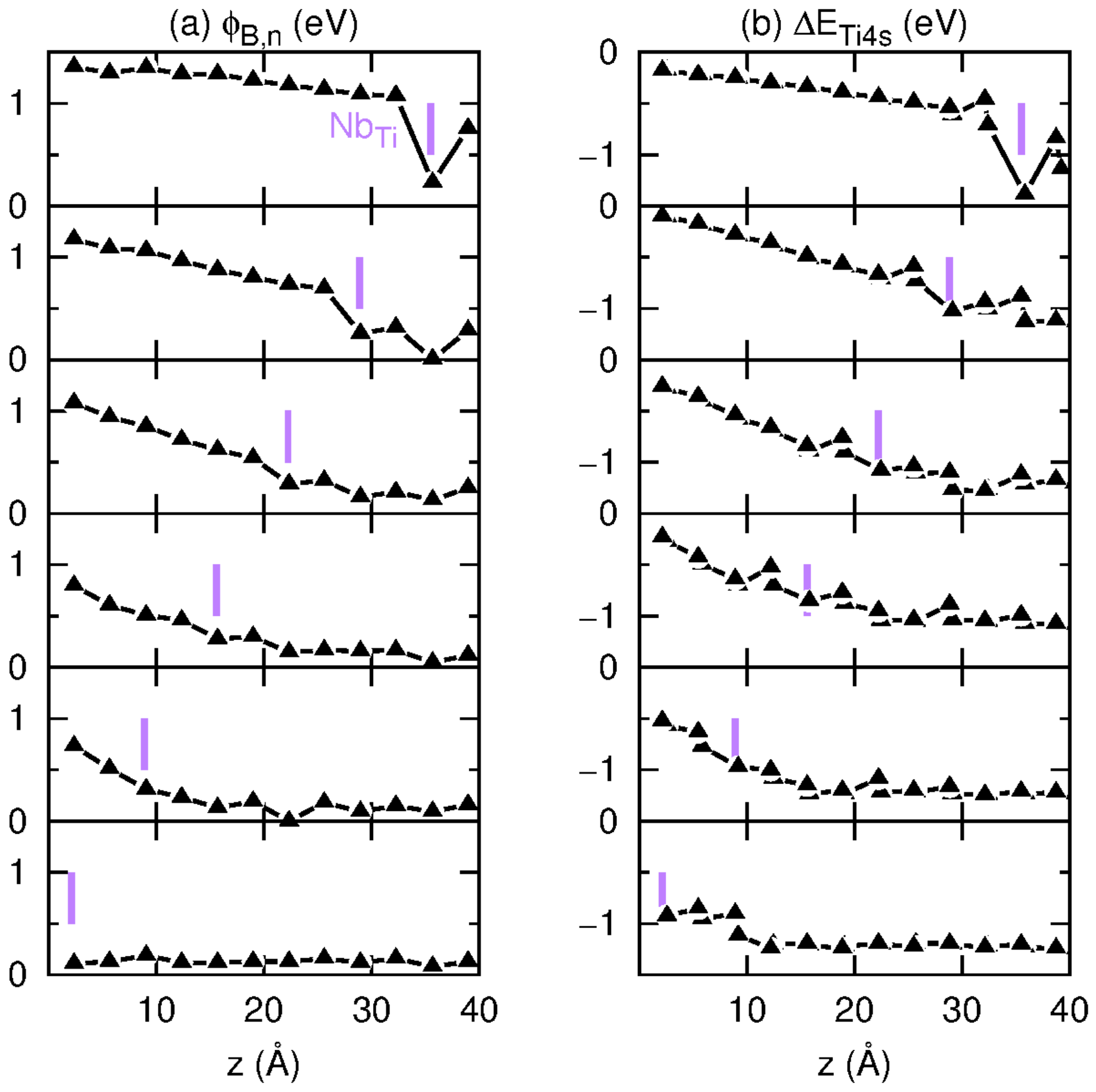

Supplementary Figure S3. The band bending in TiO_2 varied with *Nb* dopant interface distance (d_D). (a) The layer localised conduction band minimum (CBM) referred to the Fermi energy. (b) The dopant induced energy shift of the Ti 4s semi core state. The vertical bars indicate the position of Nb-dopant in each calculation. Both curves show that the TiO_2 bands shift up while approaching the interface.

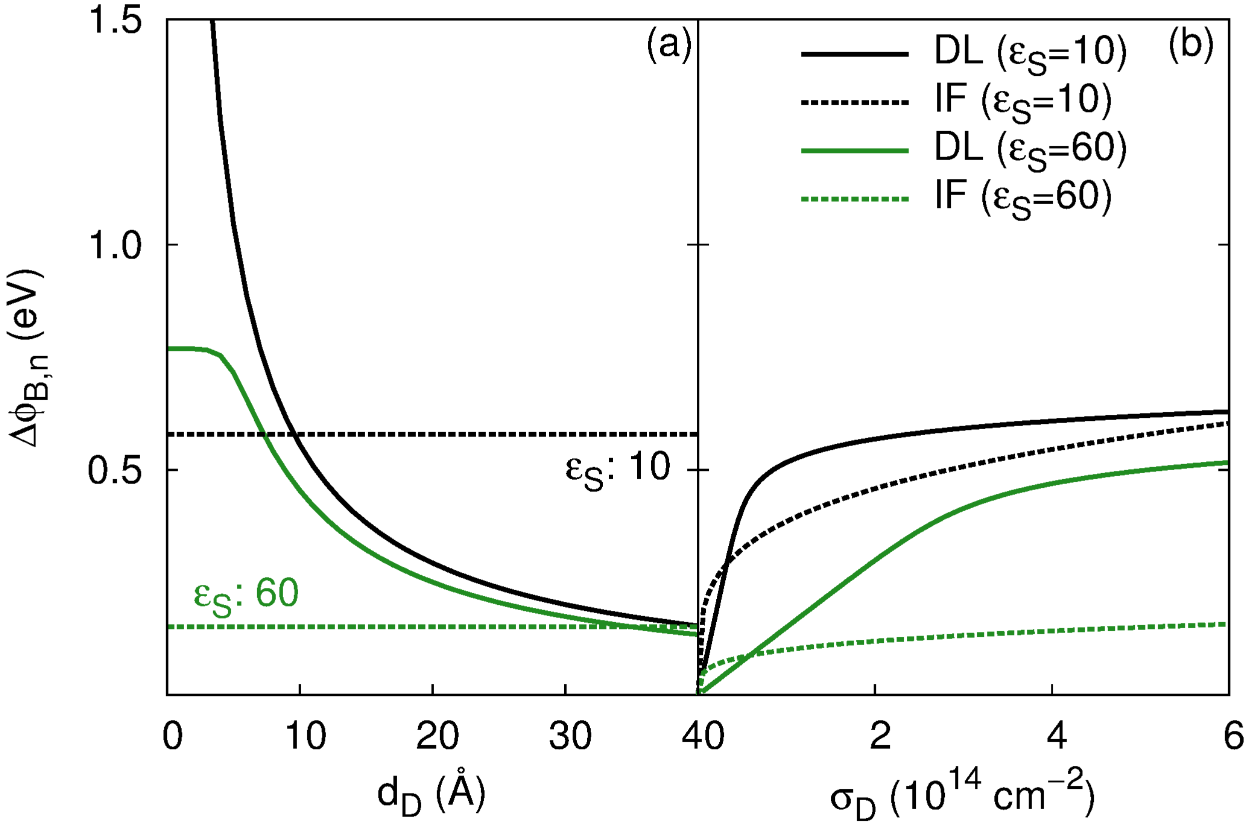

Supplementary Figure S4. The decrease of the SBH as a function of the dopant interface distance (a) and dopant areal concentration (b) using model simulations with low and high dielectric constants (ε_S). In the analysis of SBH with image force (IF) correction and deep level (DL) model, the dielectric constant ε_S was treated as a parameter. DL model with $\varepsilon_S = 10$ shows good agreement with our first-principles calculations (Fig. 4). As TiO_2 has dielectric constant of $\varepsilon_S^{1000K} = 97$ and 58 in the c and a directions,[1] we also calculated the model predicted SBH reduction $\Delta\Phi_{B,n}$ with dielectric constant of $\varepsilon_S = 60$. The uniform dopant Schottky model with IF correction (Eq. 4) is more sensitive to the dielectric constant as compared to localised DL model. With $\varepsilon_S = 60$ the SBH reduction is always less than 0.2 eV in the considered density range. And the depletion layer width expands from 10 Å($\varepsilon_S = 10$) to 25 Å($\varepsilon_S = 60$) under the dopant density of $N_D = 1.3 \times 10^{21}$ cm^{-3} .

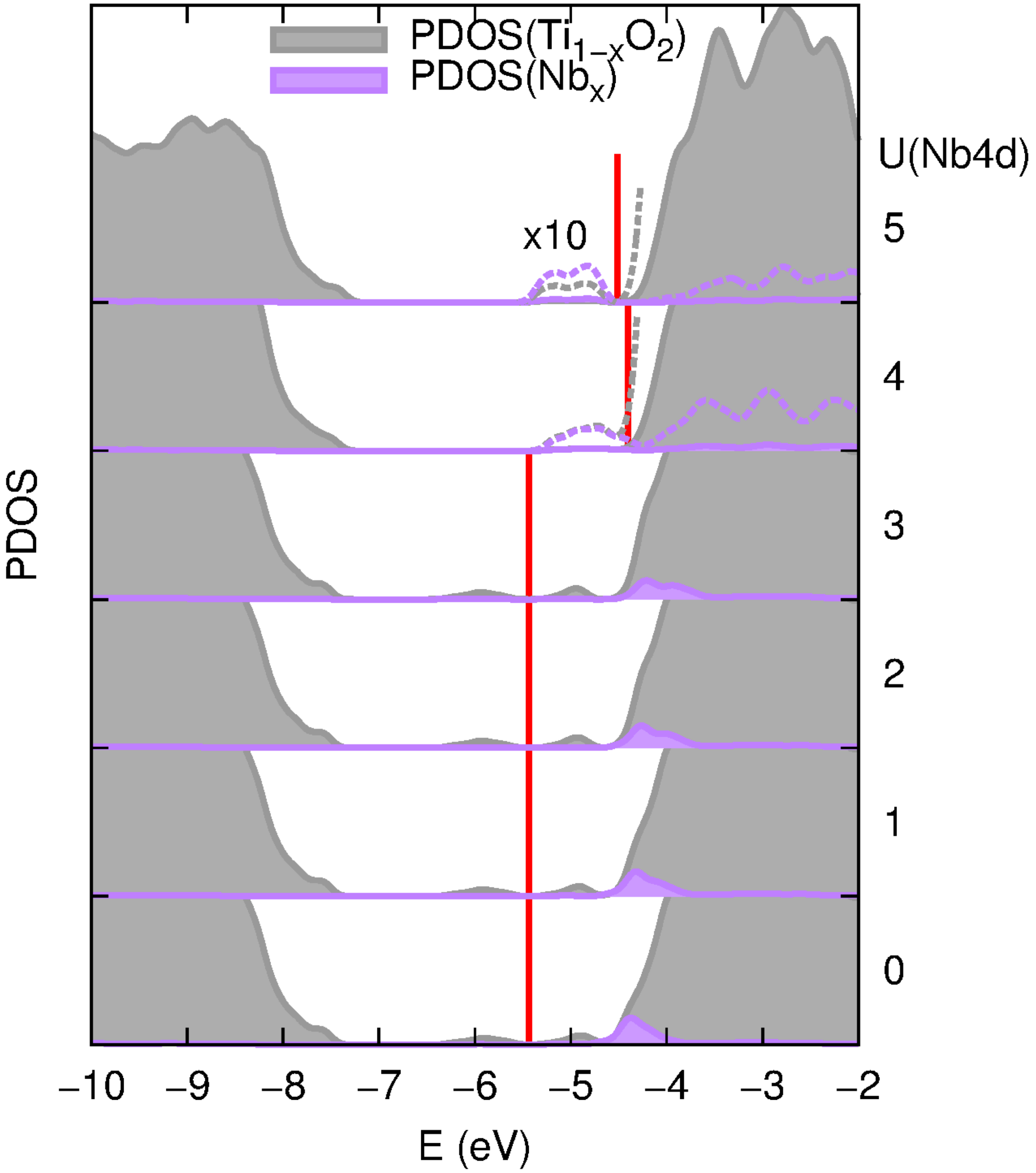

Supplementary Figure S5. The projected density of states (PDOS) of $Nb_xTi_{1-x}O_2$ on host ($Ti_{1-x}O_2$) and dopant (Nb). The $U(Ti3d)$ parameter was fixed at 10 eV and the $U(Nb4d)$ varied from 0 to 5 eV. The energy was aligned according to Ti 4s semi core orbital energy. The vertical red bars mark the Fermi energy in each calculations.

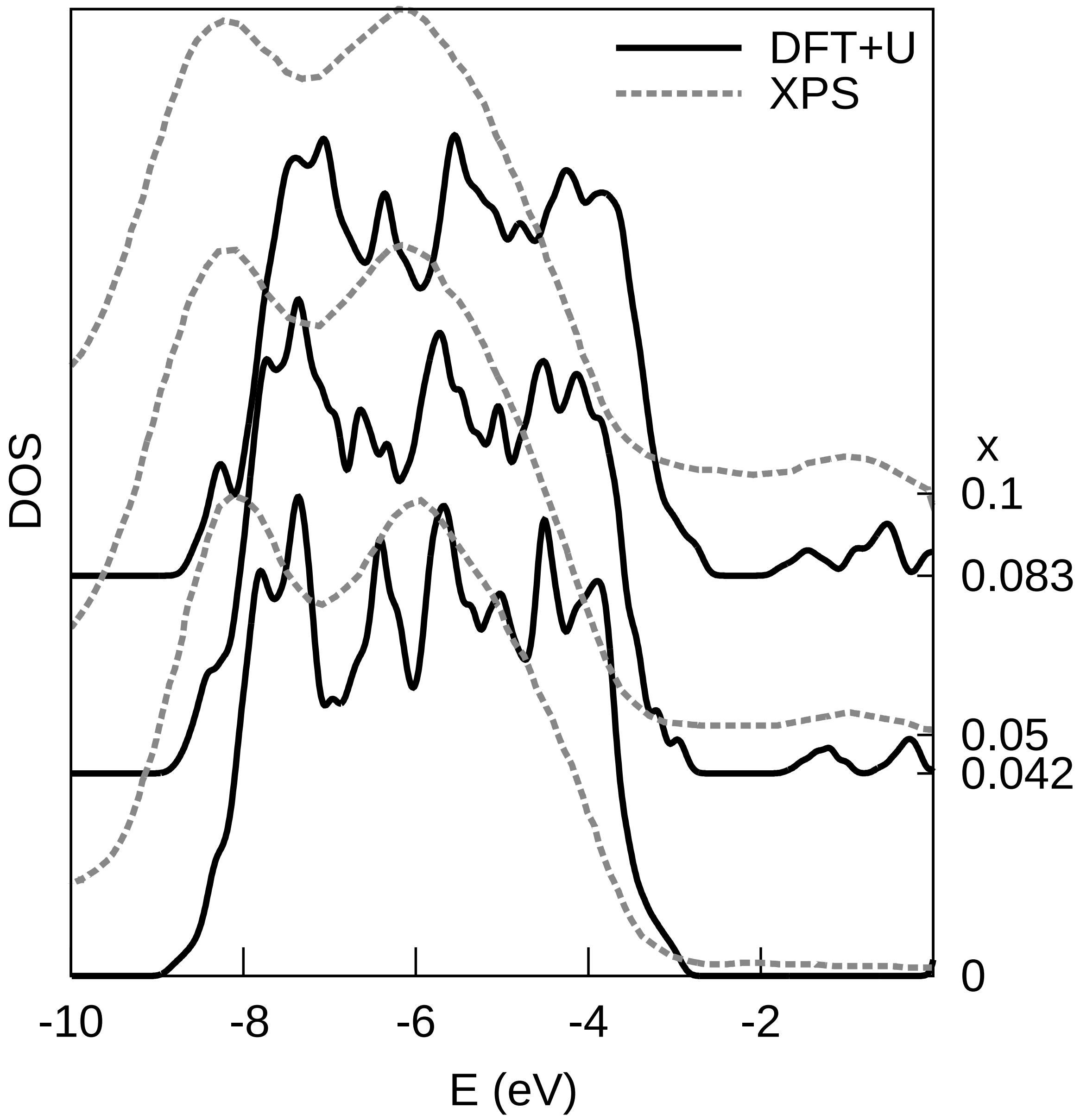

Supplementary Figure S6. The calculated density of states (DOS) using $U(Ti3d) = 10$ eV and $U(Nb4d) = 0$ eV compared with XPS experiments.[2] The energy was aligned to the conduction band minimum.

Supplementary Tables

Supplementary Table S1. The band gap (E_{gap}) in bulk rutile TiO_2 and electron affinity (χ_s) of the 12-layer rutile (110) slab. (units in eV)

		E_{gap}^{direct} ($\Gamma \rightarrow \Gamma$)	$E_{gap}^{indirect}$ ($M \rightarrow \Gamma$)	χ_s
	0	1.88	-	
	2	2.12	-	
DFT+U	4	2.39	-	
$U(Ti3d) =$	6	2.69	2.67	
	8	2.99	2.95	
	10	3.31	3.25	4.27
G_0W_0		3.41	3.34	
Exp.		3.3[3]		4.33[4]

Supplementary Table S2. The space distribution of the extra electron introduced by Nb_{Ti} on Nb atom and the two nearest neighbour Ti atoms along [001] directions. $\times 2$ indicates the charge distributions on the two nearest neighbour Ti atoms are equal.

U(Nb4d)	n_{Nb}	n_{Ti}
0	0.101	0.363×2
1	0.091	0.368×2
2	0.082	0.374×2
3	0.073	0.378×2
4	0.451	0.154×2
5	0.622	0.064×2

References

1. Parker, R. A. Static dielectric constant of rutile (TiO_2), 1.6-1060°K. *Phys. Rev.* **124**, 1719–1722 (1961). URL http://link.aps.org/doi/10.1103/PhysRev.124.1719.
2. Morris, D. *et al.* Photoemission and STM study of the electronic structure of Nb-doped TiO_2. *Phys. Rev. B* **61**, 13445–13457 (2000). URL http://link.aps.org/doi/10.1103/PhysRevB.61.13445.
3. Tezuka, Y. *et al.* Photoemission and bremsstrahlung isochromat spectroscopy studies of TiO_2 (rutile) and $SrTiO_3$. *J. Phys. Soc. Jpn.* **63**, 347–357 (1994). URL http://dx.doi.org/10.1143/JPSJ.63.347.
4. Butler, M. A. & Ginley, D. S. Prediction of flatband potentials at semiconductor-electrolyte interfaces from atomic electronegativities. *J. Electrochem. Soc.* **125**, 228–232 (1978). URL http://jes.ecsdl.org/content/125/2/228.abstract.
